# Training, experience and perceptions of point-of-care ultrasound among internal medicine trainees: Implications for training, curriculum development and service delivery

**DOI:** 10.1016/j.clinme.2025.100283

**Published:** 2025-01-22

**Authors:** Ben Joseph Probyn, Cyrus Daneshvar

**Affiliations:** a University of Plymouth, Plymouth, United Kingdom; b University Hospitals Plymouth NHS Trust, Plymouth, United Kingdom

**Keywords:** Point Of Care Ultrasound, POCUS, Ultrasound, Training, Medical Education, Postgraduate Medical Education, Medical Doctor, Physician, Internal Medicine Trainees, IMT

## Abstract

Point-of-care ultrasound (POCUS) has revolutionised modern-day medicine and has widespread utilisation throughout healthcare settings. Despite the availability of ultrasounds, there are no requirements for internal medicine trainees (IMTs) to undergo any form of POCUS training. This study aimed to explore the perceptions and experience of IMTs of POCUS. A multicentre survey was distributed via mass email to all internal medicine trainees (IMT1-ST8) across two deaneries in south-west England.

A total of 42% (213/509) IMTs completed the survey. Of these, 97% (207/213) contributed to the acute medical or general medical rota and 95% (202/213) of trainees reported performing invasive procedures on call. Training in ultrasound site selection was reported by 30% (63/213) of IMTs, and 11% (23/213) worked in a trust with accessible POCUS training. Formal POCUS accreditation was reported by 16% (34/213) of trainees. Identifying sonographic findings of common medical conditions was considered useful by 86.9% of trainees. Identifying pleural effusions being the most useful (99%, 211/213), while identifying a deep vein thrombosis was the least (87%,185/213). Higher confidence scores were reported for ultrasound-assisted as opposed to ultrasound-guided ascitic or pleural procedures, although this was not significant. On a 10-point Likert scale, most trainees highly rated POCUS training (median = 10, interquartile range 8–10). Free text responses supported POCUS training in the IMT curriculum.

We found that IMTs perceive POCUS training as highly valuable. However, few IMTs are obtaining sufficient experience or training of POCUS. Opportunities for enhanced POCUS training should be embedded in training programmes.

## Highlights

• Point-of-care ultrasound is readily accessible, affordable, portable and reliable.
• Point-of-care ultrasound can aid clinical assessment and assist procedural guidance.
• The internal medicine curriculum lacks any Point-of-care ultrasound training.
• 16% of internal medicine trainees achieve Point-of-care ultrasound accreditation.
• Internal medicine trainees highly value Point-of-care ultrasound training.

## Graphical abstract

**Figure:**
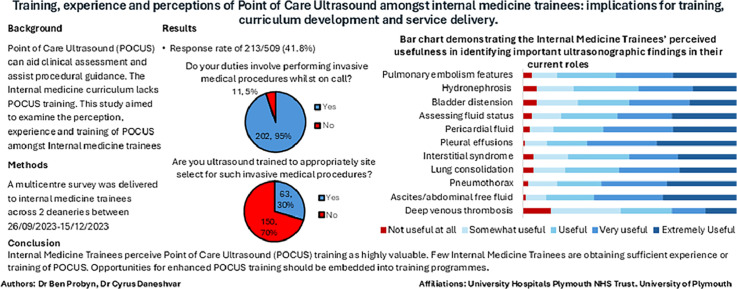

## Introduction

Point-of-care ultrasound (POCUS) has revolutionised modern-day medicine and has been branded the stethoscope of the 21st century.^1^ The portability, accessibility, affordability and reliability of POCUS in trained operators has enabled the use of POCUS to become widespread throughout healthcare settings.^2^ Moreover, another appeal of POCUS is the lack of ionising radiation. This enables POCUS to be used repetitively to reassess a patient’s condition while avoiding patient harm.^3^ Likewise, the accessibility of POCUS means that patients can avoid delays and harm incurred awaiting for departmental scans.

Diagnostically, POCUS has been used as an adjunct to the clinical history and examination. Many operators use POCUS to confirm or refute their diagnosis by examining for specific signs.^1^ Alternatively, operators may use validated algorithms, such as the BLUE protocol or eFAST (extended focused assessment with sonography for trauma), to help differentiate the cause of an acutely unwell patient.^4^^,^^5^

POCUS has also transformed diagnostic and therapeutic procedures by improving the confidence of operators at the bedside. Health professionals may use POCUS to perform diagnostic procedures such as lymph node, pleural, lung or peripheral mass biopsies in clinic. POCUS therefore enables pathways to be streamlined and reduces unnecessary delays for patients to be commenced on appropriate treatment.^6^, ^7^, ^8^

Furthermore, POCUS can be utilised to perform procedures in order to guide patient management (central venous catheterisation or arterial lines) or may be utilised to perform therapeutic procedures in their own right (paracentesis or thoracocentesis). By utilising POCUS, physicians can safely perform Seldinger techniques that may have traditionally required more invasive surgical techniques.^9^

For therapeutic procedures, POCUS can be used either be by direct or indirect guidance. Direct guidance involves visualising of equipment moving between tissue planes in real time. By this means, the operator can guide a needle into the area of interest to obtain a sample. This can be done either in or out of plane. Indirect guidance can be used immediately before performing a procedure to confirm that the operator is in an adequate position and exclude the presence of structures that would cause unfavourable results. In such a way, the operator can minimise the chance of hitting superficial anomalous blood vessels, or inadvertently penetrating nearby adjacent visceral organs.

Given the multitude of POCUS applications, POCUS has been readily adopted across specialties in community and hospital settings.^1^ Guidelines have been developed by the Royal College of Radiologists for medical and surgical specialties to achieve competency based practice in focused ultrasound.^10^ However, many royal colleges have developed their own POCUS competencies to enable trainees to gain achievable accreditation within their fields of practice. For instance, emergency medicine, critical care, respiratory medicine and acute medicine have designed their own respective POCUS curriculum.^11^, ^12^, ^13^, ^14^ Some societies have gone further, with the British Thoracic Society (BTS) creating a four-tier level of thoracic ultrasound in attempt to standardised thoracic ultrasound skills across specialties.^15^

The internal medicine curriculum highlights a number of practical skills whereby POCUS could be utilised to improve the safety profile of such procedures.^16^ Moreover, POCUS could be utilised to improve the diagnostic accuracy of doctors in training.^3^ To this end, a survey of IMTs was performed to identify which skills IMT doctors felt would be useful.

## Materials and methods

A multicentre survey was designed and distributed via mass email to all IMTs (IMT1–ST8) across two deaneries in the south-west of England (Peninsula and Severn). Follow-up emails were sent via the assistance of trust-wide postgraduate medical education centres or general medicine rota coordinators. Any responses from doctors other than IMTs were subsequently removed. Responses were collated from 26 September 2023 – 15 December 2023. Ethics approval was granted by the University Hospital Plymouth NHS Trust Clinical audit and service evaluation department reference CA_2023-24-134. Data were collected in Excel, and described using median, interquartile range (IQR) or proportions. Comparisons were made using chi-squared tests. A *p*-value of <0.05 was considered significant.

## Results

### Training and experience

A total of 213/509 (41.8%) IMTs completed the survey. This was separated into 125/213 (58.7%) IMT stage 1 trainees (IMT years 1–3) and 88/213 (41.3%) IMT stage 2 trainees (ST4+ years, higher specialty training). In total, 11 specialties were represented (acute medicine, cardiology, endocrinology, gastroenterology (including hepatology), general internal medicine, geriatric medicine, neurology, renal, respiratory, rheumatology and triple accreditation trainees). Of those polled, 207/213 (97.2%) contributed to either the acute medical take or the general medical rota and 202/213 (94.8%) stated that their duties involved performing invasive medical procedures when on call.

Although 63/213 (29.6%) of trainees reported adequate ultrasound training to identify the appropriate site for performing invasive procedures, 196/213 (92.0%) of trainees reported that they would feel more confident after further POCUS training. Local training in POCUS was available to 23/213 (10.8%) of participants. Further afield, 63/213 (29.6%) of respondents were aware of region-wide POCUS training courses and 61/213 (28.6%) of participants had attended such courses. On a 10-point Likert scale, most trainees considered POCUS training to be useful in their current role (median score = 10, IQR 8–10).

In terms of recognition, 34/213 (16.0%) of responders had formally accredited with a national award body. Most trainees accredited with award bodies with direct oversight of their specialty training. The noticeable exception was the number of respiratory trainees (n=10) accrediting with the Royal College of Radiologists (Table 1).

**Table 1:** Number of respondents who report having achieved POCUS accreditation with a national awards body, subdivided into the respective award bodies per trainee.

Are you accredited with any of the following ultrasound award bodies?		
No formal accreditation	179	
Accreditation	34	

If so, which of the below national bodies are you accredited with?	Number of accredited individuals	Specialty breakdown
British Society of Echocardiography	5	5 x cardiology trainees
British Thoracic Society (BTS)	13	11 x respiratory trainees 2 x IMT3
Royal College of Emergency Medicine	1	1 x IMT3
Royal College of Radiologists (RCR)	9	7 x respiratory trainees 2 x AIM
Society for Acute Medicine	1	1 x IMT3
The Intensive Care Society (ICS)	1	1 x cardiology
Dual accreditation (RCEM/ICS or RCR/BTS)	4	1 x IMT3, 3 x respiratory trainees

### Perceived usefulness of POCUS

**Fig. 1:**
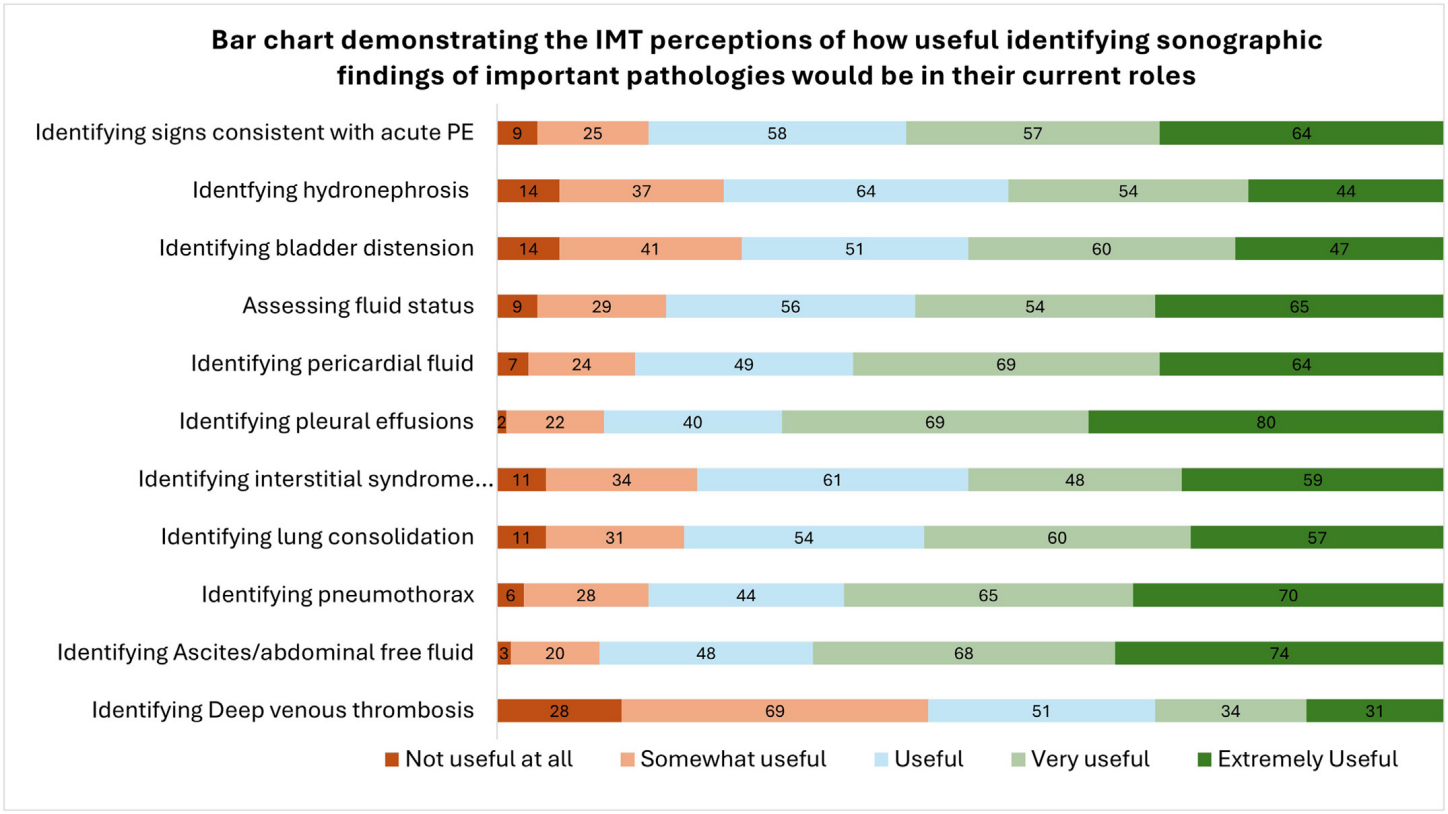
Bar chart demonstrating the IMT perceptions of how useful identifying sonographic findings of important pathologies would be in their current roles. Participant number displayed as a data label.

The perceived usefulness of ultrasonographic appearances of common medical conditions by IMTs are summarised in Fig. 1. Identifying pleural effusions was reported as the most useful sign, with 211/213 (99.1%) of participants stating that this was at least ‘*somewhat useful*’*.* Identifying ascites (210/213, 98.6%), pneumothoraces (207/213, 97.2%) and pericardial effusions (206/213, 96.7%) were also considered at least ‘*somewhat useful*’. Assessing for acute pulmonary embolism (PE) (204/213, 95.8%), fluid balance (204/213, 95.8%), lung consolidation (202/213, 94.8%), interstitial syndrome (202/213,94/8%), and hydronephrosis/bladder distension (199/213, 93.4%) was also valued. Deep vein thrombosis (DVT) identification was less frequently considered helpful (185/213, 86.9%). For any given pathological finding, less than 15% of respondents stated that learning to identify these ultrasonographic findings was not useful in their current roles.

### Ultrasound-guided procedures

**Fig. 2:**
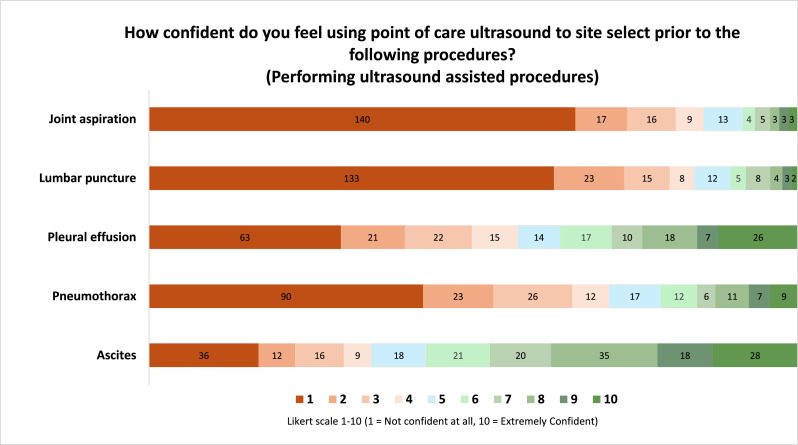
Bar chart demonstrating IMT confidence levels of performing ultrasound assisted procedures. Participant number displayed as a data label.

The survey determined the confidence level of IMTs for both ultrasound-assisted (marked directly beforehand, also known as ultrasound indirectly guided) and ultrasound directly guided procedures. For ultrasound-assisted procedures, confidence levels were low for joint aspiration, lumbar puncture and pneumothorax, with only 14/213 (6.6%), 17/213 (8%) and 33/213 (15.5%) of trainees rating a confidence level of seven or higher respectively. IMTs felt more confident using POCUS to select sites for draining ascites and pleural effusions (101/213, 47.4% and 61/213, 28.6% of trainees respectively) (Fig. 2).

**Fig. 3:**
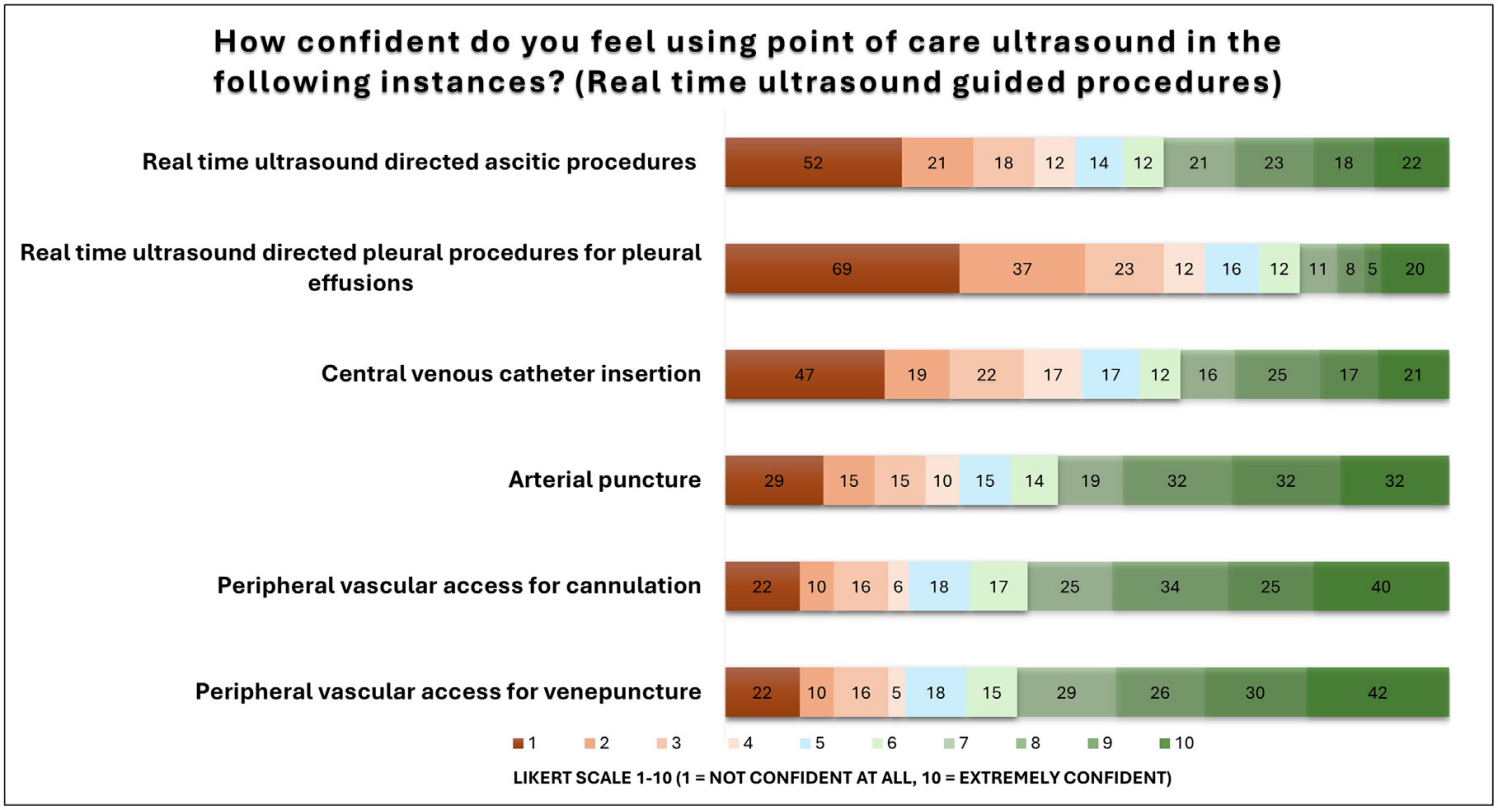
Bar chart demonstrating IMT confidence levels of performing ultrasound directly guided procedures. Participant number displayed as a data label.

In terms of ultrasound directly guided procedures, confidence was highest among IMTs for peripheral venepuncture, cannulation and arterial puncture, with 127/213, (59.6%), 124/213 (58.2%) and 115/213 (54%) respectively (Fig. 3). Confidence was lower for *real time* ascitic procedures (84/213, 39.4%), CVC (79/213, 37.1%) and pleural effusions (44/213, 20.7%). There was no statistical significance in confidence scores for ultrasound directly or indirectly guided procedures for either ascitic (X^2^ (1, n= 426)= 2.7613, *p* = 0.097) or pleural procedures (X^2^ (1, n= 426)= 3.6527, *p* = 0.056).

## Qualitative responses

The qualitative responses gave great insight into the perceived benefits and barriers of POCUS training among IMT. There was an overwhelming desire that ‘*POCUS training would be a welcome addition for GIM (IMT) training*’. Free text responses included ‘*POCUS should be a required part of the general medical curriculum*’, that POCUS training was a ‘*heavily neglected part of the medical curriculum*’ and with regard to POCUS training ‘*medics are being left behind by our colleagues..*.’. There was also concern raised that IMT doctors were ‘*expected to perform USS guided procedures without access to specialist teams*’ and a lack of POCUS training.

However, the perceived barriers of POCUS training were numerous. IMTs stated that ‘*there just aren*’*t the resources*’ to learn POCUS*.* There was a lack of ‘*formal teaching courses*’*,* ‘*lack of protected time to learn POCUS*’ and a ‘*lack of accredited supervisors*’*.* This may have contributed to the feeling there was ‘*no real encouragement to use POCUS as part of bedside assessment*’. Furthermore, those in non-procedural specialties were concerned about maintenance of POCUS skills and how they were going to ‘*keep skills up to date*’*.* A lack of POCUS training led to some trainees stating that they had become ‘s*elf-taught*’ and other colleagues stating there was a rise in medical documentation of ‘*unaccredited scans*’*.* Lastly, some IMTs felt that the UK lagged behind other countries with respect to medical physicians performing POCUS, where the perception was that this was ‘*very widespread*’*.* All free text responses can be viewed in the supplementary appendix (Table 2).

## Discussion

In this survey we found that IMTs highly rate POCUS training and want the opportunity to undergo POCUS training as part of their curriculum. We identified that many IMTs are performing POCUS without formal training, and frequently do not know how to access POCUS training. The IMTs in this survey contributed to the acute medical take and were frequently performing procedures that can be guided using POCUS. We also identified a range of common presentations whereby >85% of IMTs thought that using POCUS was either at least somewhat useful in their diagnostic assessment. However, fewer than one in three had undergone the relevant POCUS training. This is worrying, given that unregulated adoption of POCUS by untrained operators can have serious implications and is rated among the top 10 technological hazards for patient safety.^17^ As such, it is important that IMTs are appropriately trained, supervised and assessed in order to deliver POCUS. However, there are currently no training requirements for IMTs in POCUS.

Interestingly, 56.8% of IMTs stated that identifying ultrasonographic appearances of an acute PE would be very or extremely useful and this is compared to 30.5% for DVT. However, The BLUE protocol explains that an operator could incorporate a DVT scan as part of the investigations to differentiate the cause of a hypoxic patient (a rule in test).^5^ As such, it may be counterintuitive to exclude learning how to identify the ultrasonographic appearance of DVTs. Another, plausible explanation may be that participants are wary of the scanning time required to diagnose non-life-threatening medical conditions. Alternatively, the hierarchy of competence would suggest that this may represent a situation where participants are unaware of what they do not know (unconscious incompetence). For example, operators may not appreciate that the ultrasonographic findings of a DVT in a patient with hypoxic respiratory failure would be highly suggestive of an acute PE and therefore learning how to identify a DVT would be a key step in this process. Therefore, the perceived relevance of ultrasonographic findings in a POCUS curriculum design for IMTs need to be considered with caution.

We found higher confidence levels for identifying sites to drain pleural fluid or ascites than other medical procedures. This may be explained by national guidelines for pleural and large volume ascitic drainage mandating image guidance to reduce complications.^18^^,^^19^ As such, IMTs are likely to be gaining exposure and familiarity to ultrasound-guided procedures, but this may lead to a false sense of confidence in the absence of appropriate training.

Alternatively, IMTs may feel more confident in identifying the ultrasonographic appearance of free fluid. However, we previously described how < 65% of general higher specialty trainees were able to correctly identify a moderately septated pleural effusion.^20^ Moreover, it may be that an IMT feels more confident in identifying large anechoic non-septated effusions but would require further assistance when identifying more complex effusions. This would justify the tiered approach that the British Thoracic Society have created in their levels of operators descriptors for competency in thoracic ultrasound.^15^

Additionally, we found that the majority of IMTs felt confident in performing ultrasound directly guided venepuncture, cannulation and arterial puncture. However, trainees were less confident in more complex procedures such as ascitic/pleural procedures and central venous catheterisation. Real-time procedures also had lower confidence scores compared to ultrasound-assisted procedures. This reflects that real-time ultrasound-guided procedures demand greater training. As such, several curricula have been developed whereby performing POCUS to help assist in site selection is recognised as a foundation to more advanced skills of performing real-time ultrasound-guided procedures of targeted areas.^10^^,^^15^ This distinction will be important in curriculum development of IMTs, to ensure that IMTs practise within their capabilities and recognise when to escalate to specialist.

A range of free text responses by IMTs support expanding POCUS training. IMTs overwhelmingly considered that POCUS training should be a core component of the IMT curriculum. However, barriers to the implementation were numerous, including a lack of formal courses, facilitators, bedside training opportunities and protected scanning time. Some IMTs perceived POCUS utilisation by medical specialties in the UK to lag behind other developed countries. However, in the USA there are no mandatory POCUS requirements in the internal medical curriculum. LoPresti documents that the internal medicine curriculum lags behind the emergency physicians’ curriculum in the USA due to a lack of supervision, time constraints and an already demanding medical curriculum.^21^ Badejoko speculated that this may be compounded by logistical factors, a preference for other modalities, discouragement from alternative specialties and lack of perceived need of the use of POCUS among medical practitioners.^22^ Comparisons of ultrasound training and practices abroad may reveal further insight into the barriers of mass enrolment of POCUS among IMTs in the UK.

The qualitative responses also raised governance and patient safety concerns, with trainees stating that they have ‘self-taught’, and others had observed a rise in documentation of ‘unaccredited scans’. This poses patient safety concerns from operators misinterpretation of images, or from clinicians making patient management decisions based on inaccurate reports.^21^ The Royal College of Radiologists, British Medical Ultrasound Society and the Society and College of Radiographers have produced comprehensive standards to quality assure POCUS practice among specialists working independently of the radiology department.^24^, ^22^ These guidelines provide a framework to ensure that POCUS utilisation across medical specialties meets the operational, training and governance standards required for patient safety. Such guidance will provide a useful reference tool for those wishing to implement POCUS into either departments or the IMT curriculum.

In summary, we highlight the current dichotomy that exists in the training curriculum. IMTs are expected to be technically proficient at skills that mandate ultrasound training, without provisions in place to learn POCUS. Moreover, this survey highlights the strong desire and perceived benefits that IMTs feel incorporating POCUS into the IMT curriculum could bring. The current challenges in IMT training in POCUS include a lack of equipment, approved education programmes, mentorship and protected scanning time. Moreover, in order to deliver a sustainable, quality-assured POCUS programme, suitable stakeholders would be required to invest in POCUS training, curriculum, accreditation and maintenance of skills.^7^

### Limitations

The authors acknowledge that there are several limitations in this study. The response rate is 41.8% and therefore the experience of a substantial number of IMTs is not captured. Likewise, the study was performed across two deaneries in the south-west of England and may not reflect the experiences across the country. Moreover, confidence and self-reporting of accreditation were used as measure outcomes, both of which are subjective markers of performance, with no formal POCUS assessments made. Likewise, there is currently a lack of a national database to recognise POCUS-accredited providers. Lastly, although unconventional POCUS may be utilised to perform all medical procedures under real-time guidance. Certain procedures have not been polled for confidence under real-time guidance, as it is not common for IMTs to require this level of assistance (eg for joint aspirations) and alternative methodology may exist (eg fluoroscopy for difficult lumbar punctures). As this study was focused on POCUS for common findings and interventions in the general medical curriculum, confidence levels of specialties performing uncommon procedures were not included (eg pericardiocentesis by cardiologists).

## CRediT authorship contribution statement

**Ben Joseph Probyn:** Writing – original draft, Visualization, Validation, Project administration, Methodology, Investigation, Formal analysis, Data curation, Conceptualization. **Cyrus Daneshvar:** Writing – review & editing, Visualization, Supervision.

## Declaration of competing interest

The authors declare that they have no known competing financial interests or personal relationships that could have appeared to influence the work reported in this paper.

## Appendix Supplementary materials

Image, application 1
