# Supplementary material for: Training, experience and perceptions of point-of-care ultrasound among internal medicine trainees: Implications for training, curriculum development and service delivery
Source: Clin Med (Lond). 2025 Jan 22;25(2):100283. doi: 10.1016/j.clinme.2025.100283 (PMC11910704; doi:10.1016/j.clinme.2025.100283)
Supplement: Supplementary file 1 [file mmc1.docx]

**Supplementary figures and appendices.**

**Figure 1: Bar chart demonstrating the IMT perceptions of how useful identifying sonographic findings of important pathologies would be in their current roles. Participant number demonstrated as a data label.**

**Figure 2: Bar chart demonstrating IMT confidence levels of performing ultrasound assisted procedures. Participant number demonstrates as a data label.**

**Figure 3: Bar chart demonstrating IMT confidence levels of performing ultrasound directly guided procedures. Participant number demonstrates as a data label.**

| Are you accredited with any of the following ultrasound award bodies? | | |
| --- | --- | --- |
| No formal accreditation | 179 | |
| Accreditation | 34 | |
|  | | |
| If so which of the below national bodies, are you accredited with? | **Number of accredited individuals** | **Speciality breakdown** |
| British Society of Echocardiograpy | 5 | 5 x Cardiology trainees |
| British Thoracic Society | 13 | 11 x respiratory trainees  2 x IMT3 |
| Royal College of Emergency Medicine | 1 | 1 x IMT3 |
| Royal College of Radiologists | 9 | 7 x Respiratory trainees  2 x AIM |
| Society for acute medicine | 1 | 1 x IMT3 |
| The intensive care society | 1 | 1 x Cardiology |
| Dual accreditation  (RCEM/ICS or RCR/BTS) | 4 | 1 x IMT3,  3 x Respiratory trainees |

**Table 1: Demonstrates the number of respondents who report having achieved POCUS accreditation with a national awards body. This is then subdivided into the respective award bodies per trainee.**

| **Table 2: Internal Medicine Trainee views on POCUS – Unredacted free text responses** |
| --- |
| An excellent idea I have frequently wondered about - looking forward to hearing more. Please ensure updates / ongoing info is available to trainees - would not want to miss out on opportunities in the future. Thank you. |
| As a geriatric trainee, we do not do any procedures really outside of GIM on calls. These days I find that most foundation doctors and SHOs are USS trained for venous access so I have not needed to develop this skill. I am not considering attending any POCUS courses as I do not feel that I would have adequate exposure in my current role to keep skills up to date. In three years of being a med reg I have had to do only a handful of ascitic taps for which I use USS, but otherwise have not had cause to use it for anything else. It is obviously an extremely useful skill for acute medics to develop and I am fully supportive of this, but it is not something I am particulary interested in as part of my specialty. |
| Attempted to find a FAMUS course several times previously, but unable To find anyone willing or qualified to supervise the portfolio aspect. Know of several colleagues in same position. Would find ultrasound training really useful as we have good access to ultrasound, but no teaching beyond a cannula as of yet. |
| Can I attend point of care US teaching in this hospital? |
| Had CVC/art line USS experience on ITU rotation |
| Hi, In NDDH we do get informal training on US for procedure, more so on ACCS than in IMT due to more ICU ED expoosure. However our problem is doing a POCUS course with accredited supervisor to sign us off for scans is a bit pointless so most of us don't attend a course. We have 3x FUSIC trained supervisors and 1x FAMUS trained who is a once a week visitor from exeter |
| I am a bit biased as I will be undertaking EUS training soon and have organised to go to some more USS lists but do feel we should have more training in this, particularly as we are still expected to be able to do chest drains. |
| I feel that it is a heavily neglected part of the medical curriculum and feel that us medics are being left behind by our colleagues in intensive care and emergency medicine. There are very few FAMUS facilitators/supervisors in the region and no real encouragement to use POCUS as part of bedside assessment |
| I have been struggling to get access to a course in the region. Not enough dates and not enough spot available. |
| I have informally trained myself with basic abdominal ultrasound especially for ascites/liver pathology. Anything else in the body and I am out of my depth. |
| I think a POCUS course with annual refresher should be integrated into GIM training if we continue to be expected to perform USS guided procedures without access to specialist teams (out of hours for example). Adding another compulsory training to our already tight schedule may be a challenge unless it is integrated into our rota at trust level. |
| I think that Pocus should be a required part of the general medical curriculum |
| I think ultrasound training should be mandatory for medical registrar's and training should be provided if not at hospital level by the deanery itself until.each hospital has adequate staff numbers to provide internal training locally |
| I worry that even if I had some training in point of care US that I wouldn't use the skill often enough to keep up my competance (as with some of the procedural skills) |
| I would be really keen to take part in some ultrasound training, if you know of any courses available to IMTs in the Bristol and weston could you email them to us please or tell us how to book on thanks so much |
| I would like to request to arrange USG training course and it’s relative procedure in Derriford Hospital.Many Thanks |
| I'd like to take a course! |
| If there’s any POCU in Derriford, I would like to attend it please. |
| It seems insane to me that we have to be signed off as competent on procedures that require USS to do them but being able to do the USS ourselves is not required for the sign off/ part of the mandatory curriculum for IMT |
| I've done a FAMUS course but am struggling to accredit due to clinic time being needed. |
| Lack of accredited supervisors seems to limit opportunities for training. |
| not sure if trust helps with pocus training but would be great, thanx |
| Please offer to core trainees it would be very useful during oncalls |
| POCUS is extremely useful and it’s use is only becoming more widespread. Courses are expensive and difficult to access as an IMT. In Exeter they are rolling out the FAMUS course, but prioritising ACPs before junior doctors, which has ruffled a few feathers |
| POCUS should be a mandatory part of the IMT curriculum - focussed US is too important to safety and ease of procedures and rapid diagnosis |
| POCUS training would be a welcome addition for GIM training in my opinion |
| Regulation of unaccredited scans (often written in notes as unaccredited scans, how to action these reports and what to do with findings when unaccredited) |
| Seeing juniors coming through training with the ability to do US guided bloods and cannulas is great - the skill needs to be taught earlier in training (ideally pre-reg) to allow lots of time to build confidence & practice |
| Seems completely ridiculous that we are expected to do USS procedures without any training. |
| Self-taught in abdominal USS for identifying liver, spleen, gallbladder, ascites. Would appreciate gastro lean to teaching e.g. cholecystitis. |
| The more US training the better, in any job it is beneficial. |
| This would all be extremely useful and 100% should be part of our training within IMT so you can have a basic understanding as a medical registrar. It would improve patient care and diagnostic certainty. I'm pretty sure every trainee wants to be more competent with ultrasound but there just aren't the resources or trainers to do so currently. |
| This would be a really useful skill to develop during IMT |
| This would be a really useful skill to normalise among JDs on acute take. I have previously worked in Germany where POCUS us for medical rule in is very widespread and speeds up the patient journey. |
| Ultrasound is the future! stethoscopes belong in the bin |
| US guided cannulation training would be very useful for trainees on Onc/Haematology |
| While there's definitely enough options to pick up basic ultrasound skills as an IMT to confidently do venepunctures, peripheral cannulation, ascitic taps/drains, central lines, and to an extent pleural effusions, formal training is obviously going to be a significant help. |
| would appreciate if able to set up formal teaching courses with metnors. Currently, it's so difficult to find FAMUS mentors in this area. |
